# Seasonality of Acute Retinal Necrosis

**DOI:** 10.18502/jovr.v15i1.5944

**Published:** 2020-02-02

**Authors:** Alireza Hedayatfar, Maryam Ashraf Khorasani, Mehdi Behnia, Ahad Sedaghat

**Affiliations:** ^1^Eye Research Center, Rassoul Akram Hospital, Iran University of Medical Sciences, Tehran, Iran; ^2^Noor Ophthalmology Research Center, Noor Eye Hospital, Tehran, Iran

**Keywords:** Acute Retinal Necrosis Herpetic Viral Retinitis, Polymerase Chain Reaction, Seasonal Variation, Varicella-Zoster Virus

## Abstract

**Purpose:**

To study the seasonal variability in the occurrence of acute retinal necrosis (ARN) in a series of polymerase chain reaction (PCR)-positive patients.

**Methods:**

Consecutive patients clinically diagnosed with ARN and a positive PCR result of aqueous humor during a seven-year period were studied retrospectively. Patients' demographics, causative viral agent(s), and the date of disease onset were extracted from medical records.

**Results:**

Twenty eyes of 20 patients were enrolled; the mean age at presentation was 39.6 ± 14.4 (range, 6–62) years. Nine patients were female. The most common causative agent was varicella-zoster virus in 16 patients (80%), followed by herpes simplex virus in two patients (10%). The disease onset was in winter in 10 patients (50%), and the highest incidence was in February (five patients, 25%). The cumulative occurrence of ARN was significantly higher in the first half of the year (winter and spring) compared to the second half of the year (summer and fall) (*P* = 0.030). In general, seasons with a high incidence of ARN were preceded by cold seasons.

**Conclusion:**

In our series, we observed seasonal variability in the incidence of ARN, with the highest incidence during winter and spring. However, further epidemiologic studies in different geographical areas are required to elucidate the true seasonal nature of ARN.

##  INTRODUCTION 

Acute retinal necrosis (ARN) was first described in 1971 by Urayama et al as a syndrome of acute panuveitis with retinal periarteritis progressing to diffuse necrotizing retinitis and retinal detachment (RD).^[1]^ This uncommon but potentially blinding condition is usually noticed in immunocompetent hosts but occasionally among immunocompromised patients.^[2,3]^ In a nationwide survey in the UK, the estimated incidence of ARN was one case per 2 million people per year, which had been stable over one decade.^[4,5]^

ARN is caused by members of the Herpesviridae family, most commonly varicella-zoster virus (VZV) and herpes simplex virus (HSV), and occasionally Epstein–Barr virus (EBV) and cytomegalovirus (CMV).^[6,7,8]^ Although ARN is essentially a clinical diagnosis, ocular fluid polymerase chain reaction (PCR) testing has been widely used as an aid to the diagnosis and identification of the causative virus.^[9,10,11,12]^

ARN is a rapidly destructive disease. Systemic antiviral therapy is the mainstay of treatment. Corticosteroids are usually used along with antiviral agents to reduce the ocular inflammation. Antivirals hasten the remission of retinitis in the affected eye and have a protective effect on the fellow eye.^[13]^ Moreover, early laser retinopexy can reduce the risk of RD,^[14,15]^ which is a complication that is considered a major cause of poor visual outcome in ARN.^[16,17]^ Therefore, early diagnosis and prompt management are important for reducing ocular morbidity, and can potentially save a significant number of eyes from severe vision loss.

In this retrospective chart review, we observed a clustering tendency in the occurrence of ARN in specific months of the year; this observation could highlight important aspects of the epidemiology of the disease.

##  METHODS 

We retrospectively reviewed the medical records of patients diagnosed with ARN at two referral centers in Tehran from January 2011 to December 2017. Only eyes with positive aqueous PCR were included. The study protocol was approved by the Institutional Review Boards, and informed consent was obtained from the participants at the time of anterior chamber paracentesis. Data on the demographics, causative viral agent(s), and date of the start of the ocular symptoms (considered as disease onset) were extracted. ARN was clinically diagnosed based on the characteristic clinical features consisting of the presence of one or multiple peripheral foci of retinitis with rapid circumferential progression, occlusive retinal vasculitis (mainly arteritis), and prominent vitreous inflammation. Wide-field fluorescein angiography was used to confirm the occlusive nature of vasculitis and extent of retinal ischemia. A tailored laboratory work-up was performed to assess the immune status of the patients and rule-out other causes of uveitis. The average temperatures in each month from January 2011 to December 2017 were extracted based on the data provided by the WorldWeatherOnline.com in Tehran, Mehrabad Airport.

##  Technique of anterior chamber paracentesis and PCR

For aqueous sampling, anterior chamber paracentesis was performed using a 30-gauge needle attached to an insulin syringe, and 0.1–0.2 cc of aqueous humor was aspirated. The procedure was generally performed in the clinic after the examination. For patients who received an intravitreal injection of ganciclovir, both sampling and injection were performed in the operating room. The samples were kept at 2–8${}^{\circ }$C and delivered to the laboratory within 1 h and were stored at –20${}^{\circ }$C. DNA extraction was performed within one week using High Pure Viral Nucleic Acid Kit (Roche Diagnostics GmbH, Mannheim, Germany) following the manufacturer's instructions.^[18]^ Qualitative PCR was performed using the flash method by the DNA Technology Kit (DNA-Technology, Moscow, Russia).^[19]^

The results are expressed as mean ± standard error. Due to the limited number of cases, Fisher's exact test was used to compare the cumulative occurrence of ARN between the first and second halves of the year. A *p-*value < 0.05 was considered statistically significant.

**Table 1:** Demographics, causative viral agent(s), and date of disease onset in ARN cases


**Patient #**	**PCR result**	**Gender**	**Age at onset (years)**	**Laterality**	**ARN onset (month)**	**ARN onset (year)**
1	VZV	M	41	OD	6	2013
2	VZV	M	30	OS	5	2014
3	VZV	F	56	OD	1	2016
4	VZV	F	28	OS	4	2013
5	VZV	M	58	OD	10	2015
6	VZV	M	53	OS	2	2011
7	VZV	M	45	OD	5	2012
8	VZV	F	44	OU	8	2013
9	VZV	F	45	OD	6	2017
10	VZV	M	40	OD	1	2015
11	VZV	M	21	OS	2	2017
12	VZV	F	35	OS	2	2016
13	VZV	M	48	OS	1	2017
14	VZV	M	56	OS	3	2015
15	VZV	F	29	OD	3	2015
16	VZV, EBV	F	62	OS	4	2016
17	HSV	M	6	OS	2	2017
18	HSV	M	25	OD	2	2014
19	EBV	F	26	OD	3	2017
20	CMV	M	43	OD	5	2014
	
	
ARN, acute retinal necrosis; CMV, cytomegalovirus; EBV, Epstein–Barr virus; F, female; HSV, Herpes Simplex virus; m, male; OD, right eye; OS, left eye; OU, bilateral; PCR, polymerase chain reaction; VZV, Varicella-Zoster Virus						

**Figure 1:**
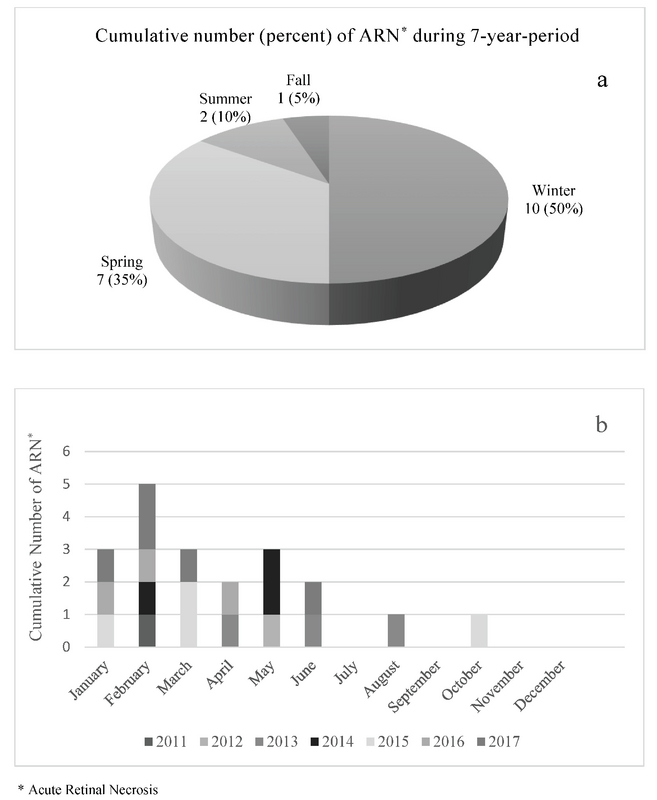
Cumulative number of cases of acute retinal necrosis in each season (a) and month (b).

**Figure 2:**
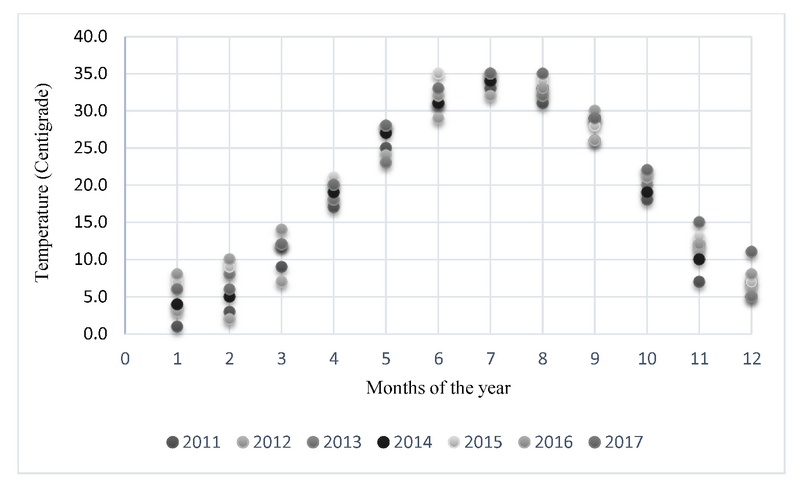
Average temperature in each month in the study period.

##  RESULTS

Twenty eyes of 20 patients were included in this study. The mean age at presentation was 39.6 ± 14.4 (range: 6–62) years. Nine patients were female. None of the patients were HIV-positive. ARN was unilateral in all patients except one [Table 1]. The patient with bilateral ARN did not consent for bilateral sampling, therefore, only the eye with more extensive involvement was sampled (positive for VZV) while the other eye was excluded from the study.

Based on the PCR results, the most common causative agent was VZV detected in 16 patients (80%), followed by HSV in 2 patients (10%; HSV-1 and HSV-2 each in one patient). CMV was detected in one patient (5%) and EBV in one eye (5%). In one VZV-positive patient, a simultaneous co-infection by EBV was present [Table 1].

In most patients (85%, 17 patients), the disease onset was in winter (ten patients, 50%) or spring (seven patients, 35%). Summer (two patients, 10%) and autumn (one patient, 5%) were the seasons with the lowest incidence [Figure 1(a)]. February was the month with the highest incidence of ARN (five patients, 25%), and July, September, November, and December were the months with the lowest incidence (no cases) [Figure 1(b)]. The cumulative occurrence of ARN was significantly higher in the first half of the year (winter and spring) compared to the second half of the year (summer and fall) (*P* = 0.030).

Figure 2 shows the average temperature in each month during the study period. The coldest month in the study period was January (mean temperature, 5.1${}^{\circ }$C), followed by February (mean temperature, 6.5${}^{\circ }$C) and December (mean temperature, 7.0${}^{\circ }$C). July (mean temperature, 34.0${}^{\circ }$C), August (mean temperature, 32.9${}^{\circ }$C), and June (mean temperature, 31.7${}^{\circ }$C) were the warmest months. The coldest season that was winter (average temperature, 7.7${}^{\circ }$C) had the highest incidence of ARN (50%). Autumn, which was the second coldest season (average temperature, 12.9${}^{\circ }$C), had the lowest incidence of ARN (5%). The high incidence rate of ARN (35%) in spring suggests a time lag between the beginning of cold seasons and an increase in the incidence of ARN.

##  DISCUSSION

In this series, there was a tendency for the occurrence of ARN during winter and spring. The seasons with a high incidence of ARN were preceded by cold seasons. While the temperature began dropping mid-autumn, the peak of ARN occurred during winter, and once the temperature started rising mid-spring, the incidence of ARN declined in summer.

Previous studies have shown patterns of seasonal variability in the incidence of infections associated with the Herpesviridae family. Varicella shows pronounced seasonality in temperate climates and most tropical climates, with a peak incidence in the cooler, drier months during winter or spring.^[20,21,22,23]^ In a study using the Connecticut statewide hospital discharge database in the pre-vaccine era, 73.2% of the varicella hospitalizations occurred during winter and spring.^[24]^ In contrast, zoster does not show any seasonal pattern in the UK, Canada,^[25]^ or Western Australia.^[26]^ Seasonal variations in the occurrence of herpetic keratitis were reported as well. In a study in Japan, a negative correlation was observed between the incidence of dendritic keratitis recurrences and temperature and 42.2% of the recurrences occurred during winter.^[27]^ Gamus found the highest rate of herpetic eye attacks during January,^[28]^ and others reported the highest incidence of herpetic keratitis in late autumn and winter.^[29]^

Seasonal cycles in infectious diseases are generally attributed to seasonal differences in weather conditions, seasonal rhythmicity in host susceptibility to infectious agents, and the virulence or prevalence of causal pathogens.^[30,31]^ The explanation of the seasonal variability in the incidence of ARN in this series would be undoubtedly complex and multifactorial. Tehran has a four-season climate. The weather is usually mild and rainy in spring, hot and dry in summer, mild to cold in autumn, but chilly and occasionally snowy in winters. Records of the average monthly temperature also showed that winter and autumn were the coldest seasons. The higher incidence rate of ARN in winter and spring suggests a temporal pattern in which the cold seasons preceded seasons with a high incidence of ARN. However, this is an assumption and cannot be confirmed using statistical methods. Administering the time series analysis, which is often used to extract possible correlations in similar situations, needs a large sample size far exceeding the number of cases included in the current study.

To the best of our knowledge, this is the first report on the concept of seasonality in ARN. We included a homogenous group of patients with PCR-proven ARN and set Tehran, a four-season city, as the reference to evaluate the probable climatological association. A limitation of the current study is the small sample size, which is due to the rarity of ARN. The second limitation is that the temperature data were collected based on records provided from a single station in Tehran (Mehrabad Airport). Considering the area of the Tehran metropolitan, a single station may not comprehensively reflect the whole climate of the source population.

The knowledge of the seasonal variability in the incidence of ARN could potentially be beneficial to ophthalmologists to expect new cases during specific months of the year and be prompt in diagnosis and treatment. However, further epidemiologic studies in different geographical areas are required to elucidate the true seasonal nature of ARN.

##  Financial Support and Sponsorship

Nil.

##  Conflicts of Interest

There are no conflicts of interest.
